# Fluorescence-Aided Identification Technique (FIT) Improves Tooth Surface Clean-Up after Debonding of Buccal and Lingual Orthodontic Appliances

**DOI:** 10.3390/jcm11010213

**Published:** 2021-12-31

**Authors:** Olivia Engeler, Oliver Stadler, Simone Horn, Christian Dettwiler, Thomas Connert, Carlalberta Verna, Georgios Kanavakis

**Affiliations:** 1 Department of Pediatric Oral Health and Orthodontics, UZB-University Center for Dental Medicine, University of Basel, 4058 Basel, Switzerland; o.engeler@unibas.ch (O.E.); oliver.stadler@unibas.ch (O.S.); simone.horn@bluewin.ch (S.H.); carlalberta.verna@unibas.ch (C.V.); 2 Department of Periodontology, Endodontology and Cariology, UZB-University Center for Dental Medicine, University of Basel, 4058 Basel, Switzerland; dettwiler@zahnarzt-weiherschloss.ch (C.D.); thomas.connert@unibas.ch (T.C.); 3 Department of Orthodontics, Tufts University School of Dental Medicine, Boston, MA 02111, USA

**Keywords:** orthodontics, debonding, composite remnants, enamel defects, FIT, fluorescent light

## Abstract

The aim of this study was to evaluate the use of fluorescence inducing light to aid the clean-up of tooth surfaces after bracket removal when using buccal or lingual orthodontic appliances. Two full sets of dental arches using extracted human teeth were assembled, with 14 teeth per arch. All teeth were bonded on their buccal and lingual surfaces. After debonding, a single blinded operator performed the tooth surface clean-up, as commonly performed in clinical practice; without the use of fluorescent light (non-FIT) and with two methods using fluorescent light to identify composite remnants on the tooth surfaces (FIT; OPAL and BRACE). Tooth surfaces were scanned before bonding and after clean-up, and the two scans were superimposed using the best-fit method. The results showed that the debonding method, type of tooth and type of tooth surface had a significant effect on the presence of composite remnants, enamel defects, and on debonding time. Contrary to the non-FIT method, there were no composite remnants after clean-up with the use of fluorescence inducing light. Clean-up time was significantly reduced on the buccal surfaces when using the FIT methods. On the lingual surfaces, the FIT methods resulted in larger enamel defects.

## 1. Introduction

After orthodontic treatment with fixed appliances and following the removal of bonded orthodontic attachments, it is important that the enamel surface is treated to its original state [1]. It is a balancing act between not damaging the enamel surface and not leaving composite remnants [2]. The latter can lead to pigmentation of the adhesive remnants caused by aging and create retentive areas that might favour biofilm accumulation, decalcification, and carious lesions [3,4].

The untreated buccal and lingual enamel surfaces differ in their tooth morphology [5]. The buccal surface with its continuous perikymata is rougher, while these horizontal ridges appear to a lesser degree or not at all on the lingual side [6]. The etching procedure leads to a dissolution of enamel prisms resulting in many pores. Those on the lingual etched surface appear smaller in diameter compared to the buccal side. Furthermore, lower debonding forces are needed on the lingual side, whereas the adhesive remnant index is lower on the buccal side [6].

There is no gold standard on how to remove composite remnants after bracket debonding, but the tungsten carbide bur remains the most preferred tool [7,8]. The clean-up with the tungsten carbide bur is fast and effective, but results in an increased enamel roughness, which requires subsequent multistep polishing [7,9].

The introduction of the fluorescence-aided identification technique provided a debonding method with high sensitivity and specificity [10,11,12,13]. It uses fluorescence inducing light to better identify composite remnants on the tooth surface. A fluorescent substance emits more visible light than it receives, making it appear brighter [14]. A near ultra-violet wavelength of 405 nm illuminates resin and differentiates it from enamel. It simplifies the detection and minimizes adhesive remnants after orthodontic debonding [11,15,16,17,18].

To the authors’ knowledge there is no previous study comparing the buccal and lingual clean-up outcomes after debonding and the impact of two different techniques used. The aim of this in vitro study was to evaluate the buccal and lingual surfaces after the clean-up procedure by assessing the amount of composite remnants, enamel loss and the time required using a conventional light source (non-FIT), and compare it to a fluorescence-aided identification technique (FIT). The null hypothesis was as follows: there is no difference between buccal and lingual clean-up regarding the presence of composite remnants, enamel loss, time required, and benefit of the technique used.

## 2. Materials and Methods

### 2.1. Tooth Model Preparation

**Figure 1:**
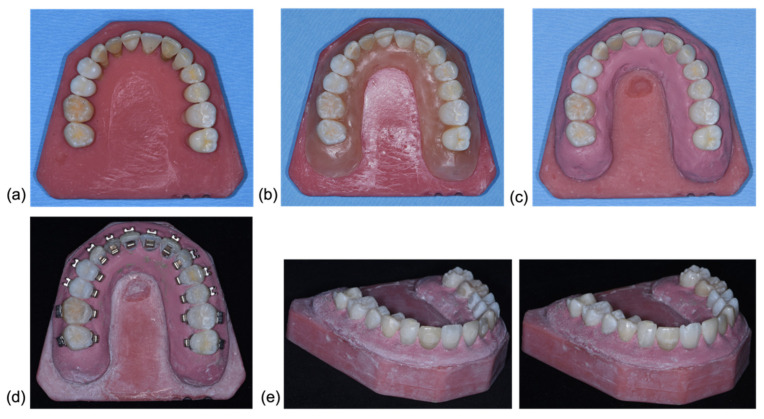
(**a**) Tooth set-up in polymer base. (**b**) Gingival mask made from wax was shaped to embed all teeth. (**c**) Wax base substituted with silicon base. (**d**) Teeth bonded buccal and lingual with their respective fixed appliances. (**e**) Upper model after bracket removal (**left**) and after surface clean up (**right**).

A total of 56 extracted human permanent teeth were collected, cleaned and stored in 0.5% chloramine-T solution at room temperature until further processing [19,20]. Two upper and two lower dental arches were produced as follows: Fourteen teeth ranging from 17 to 27 and 37 to 47 respectively were arranged in their intra-arch positions with interdental contacts mimicking a dental arch in a wax plate. They were interlocked with hot-setting-glue, and embedded in a hot polymer base (ProBase, Ivoclar Vivadent AG, Schaan, Liechtenstein). A gingiva mask was shaped with wax (BELLADI Superior Rosa, Belladi Ruscher Schleusser, Amriswil, Switzerland) and afterwards substituted with a silicone material (Finogum Premium, Fino, Bad Bocklet, Germany) (Figure 1). A digital 3D-surface-scan (inEos X5, Dentsply Sirona, York, PA, USA) of each model was performed, preoperatively—scan before bonding (T0).

**Figure 2:**
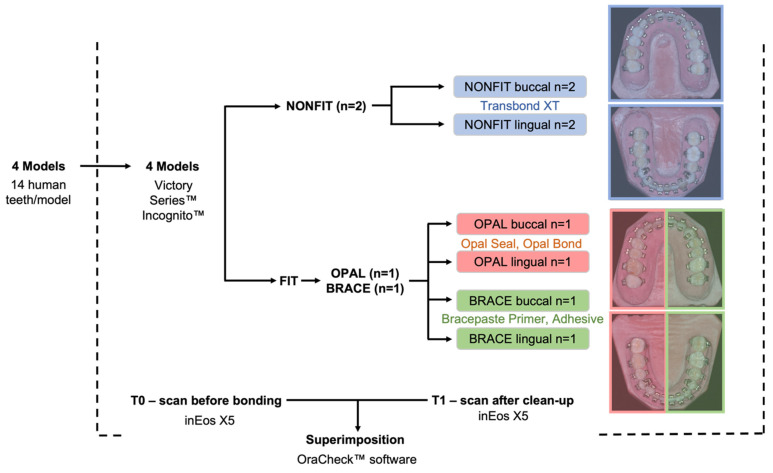
Study flow chart.

One upper and one lower dental arch formed the conventional light source Group non-FIT (*n* = 2). The other upper and lower dental arch were assigned to the fluorescence-aided identification technique (FIT; *n* = 2). The FIT group was further divided into two subgroups: Group OPAL (first and third quadrant) with a fluorescent adhesive and a fluorescent resin, and Group BRACE (2nd and 4th quadrant) with a nonfluorescent adhesive and a fluorescent resin (Figure 2).

Each tooth was bonded from buccal and lingual; buccal in a direct bonding procedure with conventional brackets (Victory Series, 3M, St. Paul, MN, USA) and lingual with customized brackets (Incognito™, 3M, St. Paul, MN, USA) with indirect bonding.

The mean surface size for the buccal bracket base was 10.93 mm^2^ (range 9.08–14.19 mm^2^) and for the lingual bracket base 19.67 mm^2^ (range 13.01–36.38 mm^2^). All the buccal and lingual tooth surfaces were etched with 35% phosphoric acid gel (Ultra-Etch; Ultradent Products, South Jordan, UT, USA) for 30 s, rinsed with water spray for 10 s and air-dried for 10 s. The prepared enamel surfaces were sealed: Group non-FIT with Transbond XT Primer (Transbond XT Primer, 3M, St. Paul, MN, USA), Group OPAL with Opal primer and sealant (Opal Seal, Ultradent) and Group BRACE with Bracepaste Primer (Bracepaste MTP Primer, American Orthodontics, Sheboygan, WI, USA).

Primers were light-cured for 5 s in all groups according to the manufacturer’s recommendation (Bluephase 20i, Ivoclar Vivadent AG, Schaan, Lichtenstein). Afterwards brackets were bonded with Transbond XT (Transbond XT, 3M, St. Paul, MN, USA) for Group non-FIT, with Opal Bond (Ultradent) for Group OPAL and with Bracepaste (Bracepaste, American Orthodontics, Sheboygan, WI, USA) for Group BRACE, respectively. First the lingual brackets were placed with the transfer jigs (Incognito™, Clear Precision Tray, St. Paul, MN, USA) consisted of two separate layers: A rigid outer layer and an inner soft layer and light cured for 20 s from the incisal, cervical, mesial and distal aspects of each bracket (Bluephase 20i, Ivoclar Vivadent AG, Schaan, Lichtenstein). After the polymerization the transfer jigs were removed. In a second step the buccal brackets were firmly placed on the prepared enamel and light cured in the same pattern as the lingual brackets.

### 2.2. Surface Clean-Up

**Figure 3:**
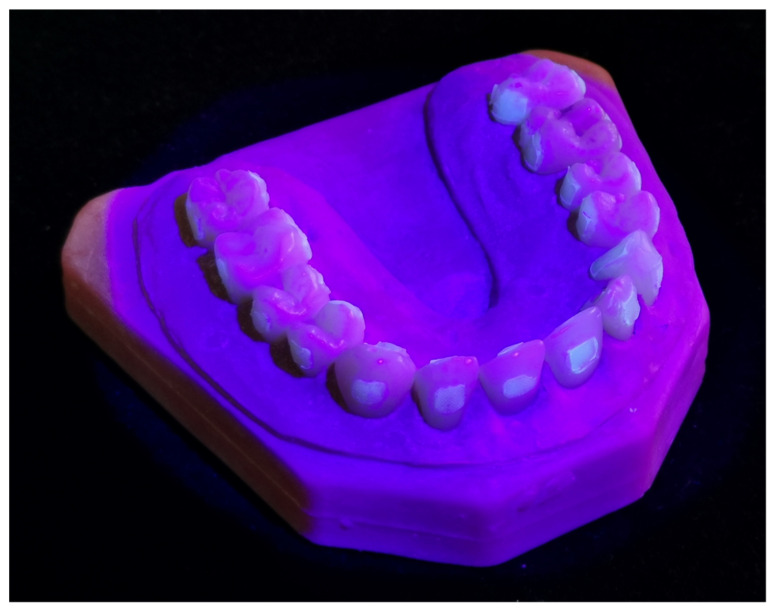
Upper typodont visualized with the FIT method, after bracket removal on the buccal and lingual surfaces.

To simulate a clinical situation, the models were temporary mounted in a dental manikin (Frasaco GmbH, Baden Württenberg, Germany) which was fixed on a dental chair (Teneo, Dentsply Sirona, York, PA, USA). The debonding procedure was performed by one right-handed operator who was not wearing glasses, had no visual impairment and had more than five years of experience. The operator was blinded regarding the purpose of the study and the measured outcomes. The Ishihara test showed neither a colour blindness nor a colour weakness. The appearance of the typodonts under fluorescence inducing light prior to surface clean-up is shown in Figure 3.

**Figure 4:**
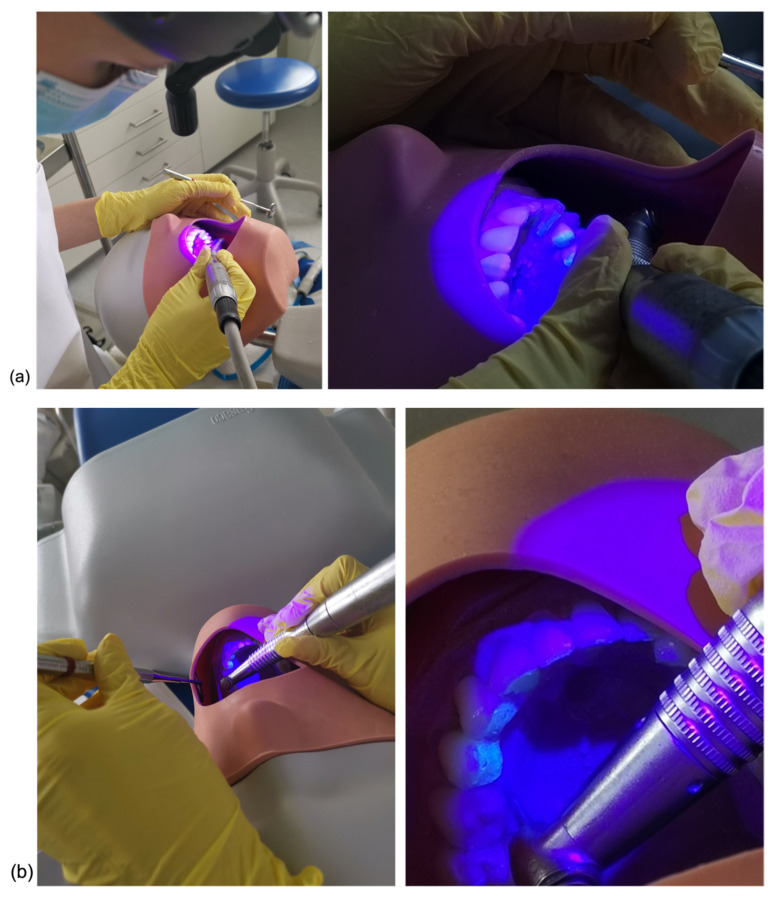
Clinical set-up for surface clean-up on the upper (**a**) and lower (**b**) dental arches.

The operator was instructed to clean the enamel surface until no visible composite remnants could be detected either buccally or lingually. To start the debonding procedure, a tungsten carbide bur (H23RA, Gebr. Brasseler GmbH, Nord-Rhine Westpahlia, Germany) mounted in a low-speed contra-angle handpiece (KaVo Master Series, Baden Württenberg, Germany) was used by applying first water cooling and then air cooling. Next, the enamel surface was polished with the silicone polishers Brownie and Greenie (Shofu, Kyoto, Japan) with water cooling. Only a dental mirror, a probe and a multifunctional syringe were allowed for detection and no magnification loops were used. The operator was asked to perform the debonding first on the non-FIT models under dental chair light (LEDview, Sirona, Hessen Germany) and then on the FIT models by using a prototype fluorescence inducing (λ = 405 ± 10 nm) headlamp (Karl Storz GmbH & Co. KG, Baden-Württenberg, Germany) The FIT prototype has been introduced and described in previous publications [10,12]. Of each model a postoperative digital 3D-surface-scan was performed—scan after clean-up (T1). The total time from start of composite remnants removal to the end of polishing was recorded in seconds with a digital stopwatch. The procedure of surface clean-up on the upper and lower dental arches can be visualized in Figure 4a,b.

### 2.3. Surface Analysis

**Figure 5:**
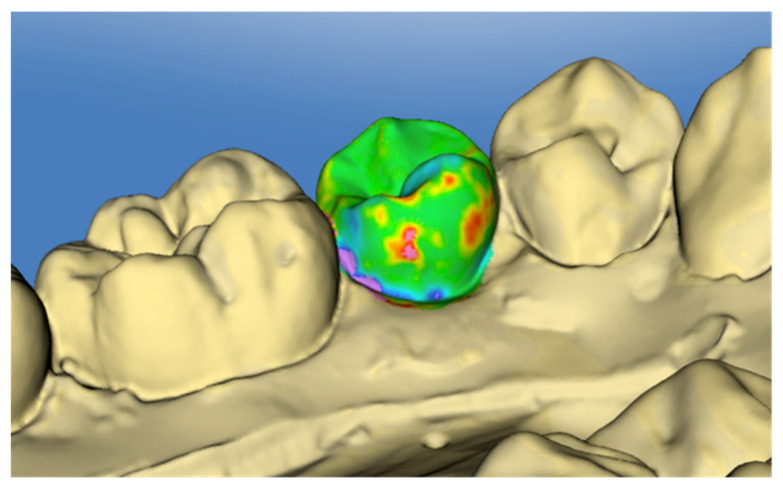
Superimposed 3D scans T0 and T1. Green represents unchanged areas; substance loss is indicated by blue and violet, excess material by yellow, red, and pink.

The OraCheck software (Version 2.13.8676, Cyfex AG, Zurich, Switzerland) was used to compare T0 and T1 scans. Pre- and post-operative digital models were first adjusted and approximated manually to aid automatic superimposition. Then, superimposition was performed separately for every tooth and the entire tooth surface was used as the superimposition area. The scan at T0 was always considered as the base scan. The superimposition areas were selected manually and the superimposition was performed automatically using the best-fit method. This automated process is performed based on an algorithm that is not provided openly by the software developer [12,21]. A total of 112 tooth surfaces were superimposed separately and analysed by color-coding to the nearest 0.01 mm (Figure 5). The linear measurements in µm were performed with the “cursor-distance” tool for composite remnant height and defect depth. For the volumetric measurements—composite remnant volume and tooth substance loss—the region of interest was selected and calculated with the “volume analysis” tool. Each tooth surface was analysed once by the same examiner.

### 2.4. Statistical Analyses

To assess the effect of various independent parameters on the severity of the enamel defects and the amount of composite remnants, as well as the time for surface “clean-up”, a multivariate regression model was developed. The dependent variables were the height and volume of composite remnants, the depth and volume of enamel defects and the time of surface “clean-up”. The predictor variables were the type of tooth surface, the type of tooth and the method for surface “clean-up”. The normality of the dependent variables was assessed with histograms and the accuracy of the regression model was tested post-hoc by plotting the standardised residuals of every dependent variable against its predicted values. Individual associations between variables were evaluated with between-subjects effect sizes and parameter estimates. Also, based on the normality of data, group comparisons were also performed with the appropriate parametric or non-parametric tests. For all analyses, a type-1 error of 5% was accepted.

## 3. Results

### 3.1. Error of the Method

The error of the method was not calculated because after clean-up of the tooth surfaces and assessment of the composite remnants and the enamel deficits, the model teeth could not be used again for a repetition of all steps. However, all procedures were performed by a single, blinded examiner with more than five years of practicing orthodontics. In addition, debonding was performed in a standardized manner and according to well accepted guidelines that allow for consistent surface clean-up after bracket removal. The same methodology has been used in a previous investigation where the inter-operator error was assessed and was found to be acceptable [11].

### 3.2. Regression Assumption Testing

The variables related to composite remnants (height and volume) were not normally distributed. However, they were included in the regression model because they were primary outcomes in this investigation. The plots of the standardized residuals against the predicted values for every dependent variable revealed that the regression model is not robust for “composite remnants” and thus the respective predictive values should be interpreted with caution. (Figure S1a–e). Nevertheless, the combined effect of the predictors on the independent variables was highly significant (η^2^ = 0.883; *p* < 0.001), and thus the null hypothesis of the investigation can be rejected. Individual parameter effects are presented below. Group means and standard deviations for each dependent variable are presented in Table S1 (Supplement).

### 3.3. Composite Remnants

**Figure 6:**
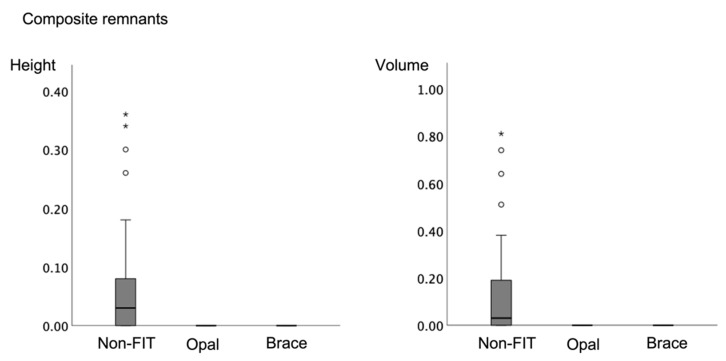
Composite remnant height (**left**) and composite remnant volume (**right**) values according to clean-up method. Both FIT methods resulted in no composite remnants. (Height in mm; Volume in mm^3^). (Note: *: Extreme outliners (Q1-(IQRx3)) and °: Mild outliers (Q1-(IQRx1.5))).

**Figure 7:**
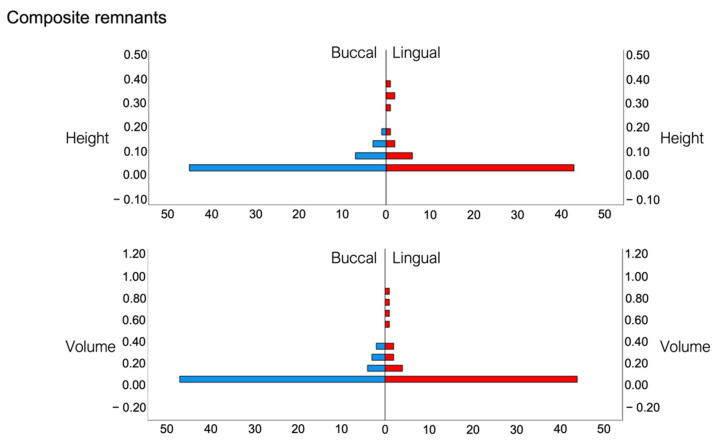
Composite remnant height (**top row**) and composite remnant volume (**bottom row**) values on the buccal (blue columns) and lingual (red columns) surfaces. (Height in mm; Volume in mm^3^).

With the FIT groups OPAL and BRACE, no composite remnants were detected either on the buccal or on the lingual tooth surfaces. Due to the lack of normal distribution in the values for composite remnant height and volume, non-parametric tests were used for group comparisons. Evaluations of the height and volume of the composite remnants according to the applied method were done with the Kruskal-Wallis test for independent samples, and revealed significant differences between the FIT groups and the non-FIT group (*p* < 0.001). Comparisons between lingual and buccal surfaces were performed with the Mann-Whitney U-test and showed that there was no difference between lingual and buccal surfaces regarding the height and volume of the composite remnants (P_height_ = 0.191 and P_volume_ = 0.177). These results are displayed in Figure 6 and Figure 7, respectively.

### 3.4. Enamel Defects

**Figure 8:**
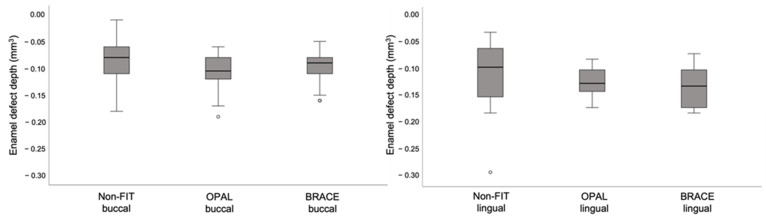
Box-and-whisker plot showing enamel defect depth for buccal (**left**) and lingual (**right**) in the three groups. (Note: °: Mild outliers (Q1-(IQRx1.5))).

**Figure 9:**
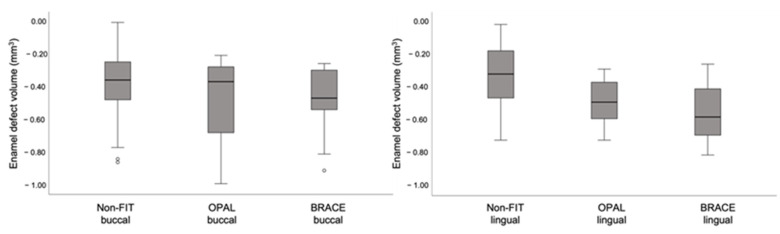
Box-and-whisker plot showing enamel defect depth for buccal (**left**) and lingual (**right**) in the three groups. (Note: °: Mild outliers (Q1-(IQRx1.5))).

The depth of enamel defects after surface clean-up was associated with the method used (η^2^ = 0.036; *p* = 0.046), the type of tooth (η^2^ = 0.064; *p* = 0.008) and the tooth surface (η^2^ = 0.067; *p* = 0.006), although the effect sizes were small. In regard to the volume of the resulting enamel defects, they were related only to the method used (η^2^ = 0.116; *p* < 0.001) and not to the type of tooth (η^2^ = 0.034; *p* = 0.052) or tooth surface (η^2^ = 0.000; *p* = 0.963). Due to the effect of the tooth surface on enamel defects, the buccal and lingual surfaces were also evaluated separately to assess the association between tooth surface and clean-up method. On the buccal side, there was no difference between the non-FIT method and the FIT methods in the depth (*p* = 0.154) or the volume (*p* = 0.314) of the enamel defects. However, on the lingual side the volume of the enamel defects was significantly larger in the FIT groups (*p* < 0.001), while the enamel defect depth remained similar (*p* = 0.243). These differences are also presented in the box-plots in Figure 8 and Figure 9 and in the Table S1 (Supplement).

### 3.5. Clean-Up Time

Surface clean-up time per tooth was significantly affected by the type of method used (η^2^ = 0.366; *p* < 0.001) and the tooth surface (η^2^ = 0.725; *p* < 0.001) but was not related to the type of tooth (η^2^ = 0.000; *p* = 0.861). The subgroup analysis for buccal and lingual surfaces revealed that on the buccal surface, the FIT methods resulted in a significantly faster clean-up of the tooth surface (*p* < 0.001). The opposite was true on the lingual surface, where the FIT methods resulted in a significantly slower clean-up time compared to the non-FIT method (*p* < 0.001). On the buccal side, the FIT methods were more than 30” faster than the non-FIT one, while on the lingual side the difference was only a few seconds (Table S1) (Supplement).

## 4. Discussion

This in vitro study evaluated the effect of using fluorescence inducing light (FIT method) to clean up the tooth surface after debonding of lingual or buccal orthodontic appliances. It was found that clean-up method, tooth type and tooth surface were significantly associated to the composite remnants on the tooth surface, the produced enamel defects and the time required for clean-up.

To assess the aims of the study, an in-vitro methodology was applied. Full arch models of extracted human teeth were mounted on a dental manikin and were used for both the buccal and lingual surface clean-up in order to simulate clinical practice as much as possible, particularly regarding the limitations stemming from the patient’s head position and the access to the tooth surfaces. Although in-vitro investigations do not perfectly resemble clinical situations, they are helpful in standardizing study conditions and controlling for confounding factors related to individual patient biology and/or behavior. Similar experimental methodology has been followed in previous studies assessing bonding strength and microleakage of novel flash-free bracket systems [22,23], providing reliable clinical information. Research evaluating the clinical properties of orthodontic wires is also conducted in a controlled in vitro design, leading to conclusions that help clinicians select appropriate wires in their clinical practice based on their performance rather than their advertised features that are often misleading [24,25,26,27].

A direct digital 3D-surface scan was used to scan the typodonts before bonding (T0) and after surface clean-up (T1) to eliminate any possible intermediate steps required by the scanning of plaster casts and therefore reduce the risk of imprecisions [28]. Also, single tooth superimpositions were performed to assess changes. This was done because the scanning precision for a single tooth is the highest and declines from quadrant to full arch [29,30,31] and because of the lack of stable superimposition areas on the typodonts, as compared to scans of actual dental arches [32]. This methodology has been used previously and has shown good applicability [11].

In the present study, composite removal was as successful on the buccal as on the lingual surfaces, with no difference in height or volume of composite remnants, however, the volume of resulting enamel defects was significantly larger on the lingual tooth surfaces. This difference could be related to the experience of the orthodontist performing the surface clean-up. The lingual appliance was introduced in 1979 by Fujita [33] and is thus less years in the orthodontic market. The appliance is also not used as often as the buccal appliance and therefore orthodontists are mostly trained to work on the buccal tooth surface. In addition, the grooves, pits and fissures of the lingual tooth surface are less accessible, creating an additional challenge in the removal of composite remnants without damaging the enamel surface [34,35]. This is particularly true on the lingual surfaces of the mandibular premolars and molars, whose morphology presents significant variation [36]. Also, it can be assumed that the differences found here will be more noticeable in vivo due to the presence of saliva and tongue movements, although every effort was made to simulate a real clinical setting.

The assessment of using fluorescence inducing light (FIT methods) for surface clean- up after bracket debonding provided mixed results in the present investigation. With regard to composite remnants, both FIT methods (OPAL and BRACE) were found to be 100% successful and significantly superior to the non-FIT method and led to complete removal of all composite remnants on the buccal and lingual tooth surfaces. The non-FIT method resulted in an average volume of composite remnants of 0.07 mm^3^ on the buccal surfaces and 0.17 mm^3^ on the lingual ones. There are no comparable studies regarding the clean-up of lingual tooth surfaces; however Ryf et al. tested various clean-up techniques on buccal tooth surfaces in-vitro and reported that when a tungsten carbide was used in combination with silicon polishers (Brownie and Greene), there are 0.11 mm^3^ composite remnants left on average [2]. This is in agreement with the present results, suggesting that this method is clinically reproducible.

Nevertheless, regarding the resulting enamel defects after surface clean-up, the FIT methods led to a significantly larger volume of enamel defects on the lingual tooth surfaces. (OPAL 0.49 mm^3^, BRACE 0.56 mm^3^) than the non-FIT method (0.34 mm^3^) There were no differences between methods on the depth of the enamel defects, nor were there any differences in the buccal tooth surfaces. In the study by Ryf et al., average volume of enamel loss was 0.22 mm^3^, as compared to 0.38 mm^3^ in this study (buccal surface, non-FIT method) [2]. This can be attributed to the individuals performing the surface clean-up. The use of a tungsten carbide bur is operator dependent, and incorrect handling can cause enamel damage, especially in cervical areas and line angles [37]. Previous studies have shown that the conditioning of the enamel with 35% phosphoric acid gel leads to a bonding infiltration of 10–20 µm into the enamel of human teeth, depending on the etching time [38,39]. Longer etching time increases the retentive enamel surface and the absolute enamel loss also increases [38]. A complete removal of this interface is not possible without damaging the enamel surface. With the FIT method the infiltrated enamel becomes visible and gets removed during the clean-up, thus leading to larger defects. Furthermore, the base of the lingual brackets was 1.8 times larger than the one of the buccal brackets, which could also be related to the larger volume of enamel defects after surface clean-up. Despite the bracket base being an important factor, this difference is also seen clinically, because lingual appliances are often custom made and have a much larger base than buccal ones. It can thus be assumed that regardless of the method used, enamel loss is more probable when using a lingual appliance due to factors related to the characteristics of the appliance (bracket base) or the morphology of the lingual surfaces, as described earlier.

Clean-up times were significantly increased for the non-FIT method on the buccal tooth surfaces, with the OPAL and BRACE methods providing a faster surface clean-up by 27.5 and 40.2 s per tooth, respectively. This finding is in agreement with previous studies evaluating the use of a fluorescence-aided technique for composite removal [11,18]. On the lingual surfaces, the results were the opposite, with the FIT methods leading to a slower surface clean-up by a few seconds. As mentioned before, this could be related to the larger bracket base of the lingual appliances. It is likely that, when using fluorescence inducing light, the operator took more time to perform a diligent surface clean-up, as instructed. This is also supported by the observation that on the buccal surfaces, the differences in composite remnants (volume and height) between FIT and non-FIT methods were less than half when compared to the differences on the lingual surfaces.

There is a large variety of clean-up methods, and thus it is difficult to perform comparisons regarding the time needed for each one [1,2,40]. The number of steps increases the mean time for surface clean-up when three or more steps are required [2]. The clean-up procedure used here included a tungsten carbide bur, which is the most commonly used, in combination with the silicone polishers Brownie and Greenie [7]. The addition of t this polishing step provides satisfactory surface clean-up. The only directly comparable study to the present one was conducted by Ryf et al., who used an identical clean-up procedure, and their results are directly comparable to our non-FIT buccal measurements [2]. In their study, 121.4 s were needed per tooth for a complete surface clean-up, significantly more than the 91.1 s reported here. Time needed for composite removal is significantly dependent on the experience and skills of the operator, thus the differences between studies are expected and mirror the differences that also exist in actual clinical practice [41].

## 5. Limitations

The present study is limited by the fact that it was conducted in vitro. Although the methodology used simulated actual clinical practice as much as possible, there are factors that would have influenced the results if the study was performed in vivo. Therefore, the results should be interpreted within the context of the applied methodology.

Also, all study procedures were performed by a single operator, which could have introduced bias as the entire process is operator dependent. As shown previously, when the same methodology is used there might be differences between operators, primarily in the time needed to remove the composite remnants [12] However, the purpose the study was to compare the efficiency of the techniques when used in clinical practice and within its limitations it provides useful information for clinicians who are interested in trying the FIT method for the removal of orthodontic appliances.

The use of loops could have improved the results of both techniques, because loops facilitate adhesive removal after orthodontic debonding [42,43]. However, most orthodontists do not use loops in clinical practice and therefore we avoided their use in this study in order to be able to better simulate the most commonly performed procedure for adhesive removal.

Finally, the results of this investigation may have been influenced by the materials used to bond the fixed appliances on the tooth surfaces. A choice of different bonding materials could have resulted in different composite remnants after debonding. This could have an effect on the time needed to perform all study procedures.

## 6. Conclusions

Both FIT methods resulted in complete composite removal on the buccal and lingual tooth surfaces, in contrast with the non-FIT method where some composite remnants were always present. When using lingual orthodontic appliances, using fluorescence inducing light for composite removal can lead to larger enamel defects compared to using the traditional method of surface clean-up. The application of fluorescence inducing light for composite removal after debonding significantly reduces surface clean-up time when buccal orthodontic appliances are used.

## Data Availability

Publicly available datasets were analyzed in this study. These data can be found at doi: 10.5281/zenodo.5810698.

## Supplementary Materials

The following supporting information can be downloaded at: https://www.mdpi.com/article/10.3390/jcm11010213/s1, Table S1: Means and standard deviations of all dependent variables according to clean-up method and tooth surface. Note the complete absence of composite remnants in the Opal and Brace groups (in bold); Figure S1: Plots of the standardized residuals against the predicted values for every dependent variable in the regression model.
