# Supplementary material for: Fluorescence-Aided Identification Technique (FIT) Improves Tooth Surface Clean-Up after Debonding of Buccal and Lingual Orthodontic Appliances
Source: J Clin Med. 2021 Dec 31;11(1):213. doi: 10.3390/jcm11010213 (PMC8746233; doi:10.3390/jcm11010213)
Supplement: Supplementary file 1 [file jcm-11-00213-s001.zip › jcm-1461450-supplementary.pdf]

Journal of
Clinical Medicine

Supplementary Material

Fluorescence-Aided Identification Technique (FIT) Improves Tooth Surface Clean-Up after Debonding of Buccal and Lingual Orthodontic Appliances

Olivia Engeler [1], Oliver Stadler [1], Simone Horn [1], Christian Dettwiler [2], Thomas Connert [2], Carlalberta Verna [1] and Georgios Kanavakis [1,3,*]

1 Department of Pediatric Oral Health and Orthodontics, UZB, University Center for Dental Medicine,
University of Basel, 4058 Basel, Switzerland; o.engeler@unibas.ch (O.E.);
oliver.stadler@unibas.ch (O.S.); simone.horn@unibas.ch (S.H.);
carlaberta.verna@unibas.ch (C.V.)
2 Department of Periodontology, Endodontology and Cariology, UZB-University Center for Dental Medicine, University of Basel, 4058 Basel, Switzerland; dettwiler@zahnarzt-weiherschloss.ch (C.D.);
thomas.connert@unibas.ch (T.C.)
3 Department of Orthodontics, Tufts University School of Dental Medicine, Boston, MA 02111, USA
* Correspondence: georgios.kanavakis@unibas.ch

Table S1. Means and standard deviations of all dependent variables according to clean-up method and tooth surface. Note the complete absence of composite remnants in the Opal and Brace groups (in bold).

Variable	Method		Mean	Standard Deviation
Composite remnant height (mm)	Non-FIT (*n* = 56)	buccal (*n* = 28)	0.04	0.05
		lingual (*n* = 28)	0.08	0.11
		total	0.06	0.085
	Opal (*n* = 28)	buccal (*n* = 14)	**0.00**	**0.00**
		lingual (*n* = 14)	**0.00**	**0.00**
		total	**0.00**	**0.00**
	Brace (*n* = 28)	buccal (*n* = 14)	**0.00**	**0.00**
		lingual (*n* = 14)	**0.00**	**0.00**
		total	**0.00**	**0.00**
Composite remnant volume (mm^3)	Non-FIT (*n* = 56)	buccal (*n* = 28)	0.07	0.11
		lingual (*n* = 28)	0.17	0.24
		total	0.12	0.19
	Opal (*n* = 28)	buccal (*n* = 14)	**0.00**	**0.00**
		lingual (*n* = 14)	**0.00**	**0.00**
		total	**0.00**	**0.00**
	Brace (*n* = 28)	buccal (*n* = 14)	**0.00**	**0.00**
		lingual (*n* = 14)	**0.00**	**0.00**
		total	**0.00**	**0.00**
Enamel defect depth (mm)	Non-FIT (*n* = 56)	buccal (*n* = 28)	-0.08	0.04
		lingual (*n* = 28)	-0.11	0.06
		total	-0.09	0.05
	Opal (*n* = 28)	buccal (*n* = 14)	-0.11	0.04
		lingual (*n* = 14)	-0.12	0.03
		total	-0.11	0.03
	Brace (*n* = 28)	buccal (*n* = 14)	-0.10	0.03
		lingual (*n* = 14)	-0.13	0.04
		total	-0.12	0.04
Enamel defect volume (mm^3)	Non-FIT (*n* = 56)	buccal (*n* = 28)	-0.38	0.22
		lingual (*n* = 28)	-0.34	0.20
		total	-0.36	0.20
	Opal (*n* = 28)	buccal (*n* = 14)	-0.48	0.26
		lingual (*n* = 14)	-0.49	0.14
		total	-0.49	0.21
	Brace (*n* = 28)	buccal (*n* = 14)	-0.47	0.20
		lingual (*n* = 14)	-0.56	0.18
		total	-0.52	0.19
Clean-up time (seconds/tooth)	Non-FIT (*n* = 56)	buccal (*n* = 28)	91.11	0.00
		lingual (*n* = 28)	103.89	0.00
		total	97.50	6.45
	Opal (*n* = 28)	buccal (*n* = 14)	63.57	0.00
		lingual (*n* = 14)	110.50	0.00
		total	87.03	23.89
	Brace (*n* = 28)	buccal (*n* = 14)	50.86	0.00
		lingual (*n* = 14)	107.86	0.00
		total	79.36	29.02

Figure S1: Plots of the standardized residuals against the predicted values for every dependent variable in the regression model.

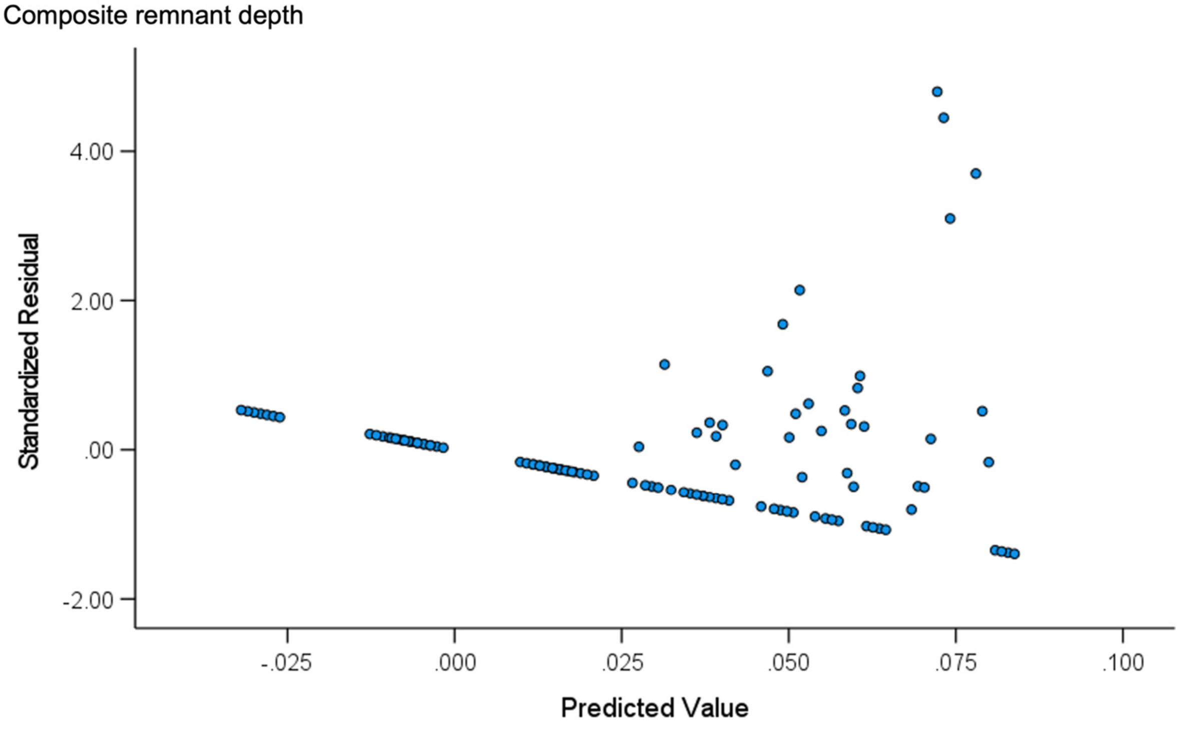

(**a**)

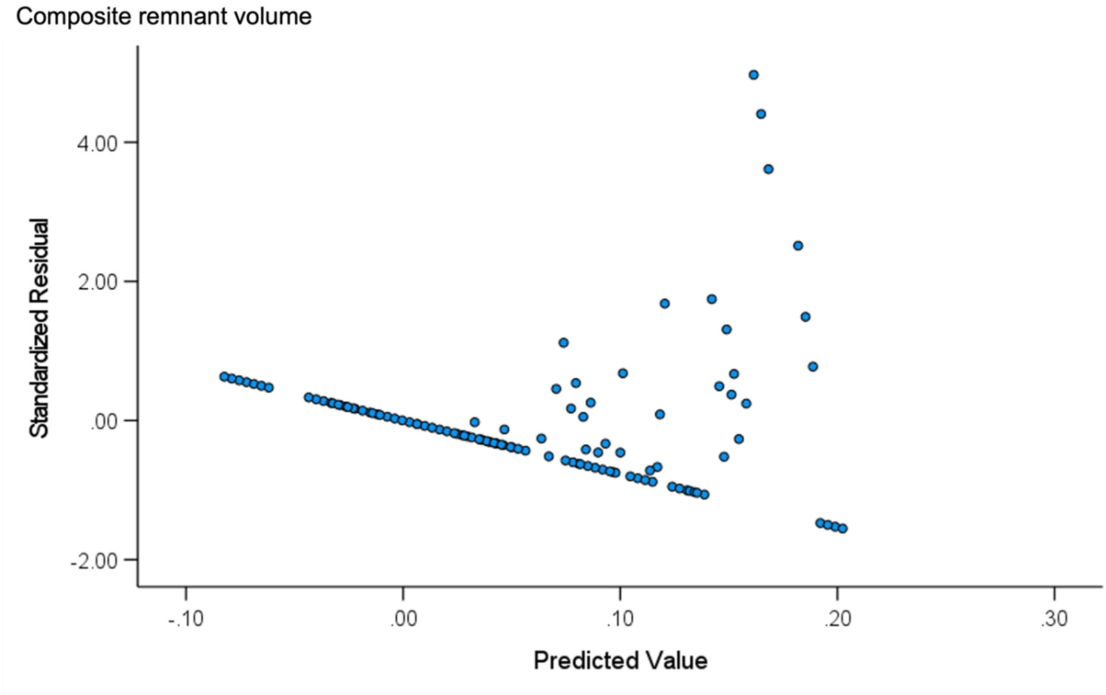

(**b**)

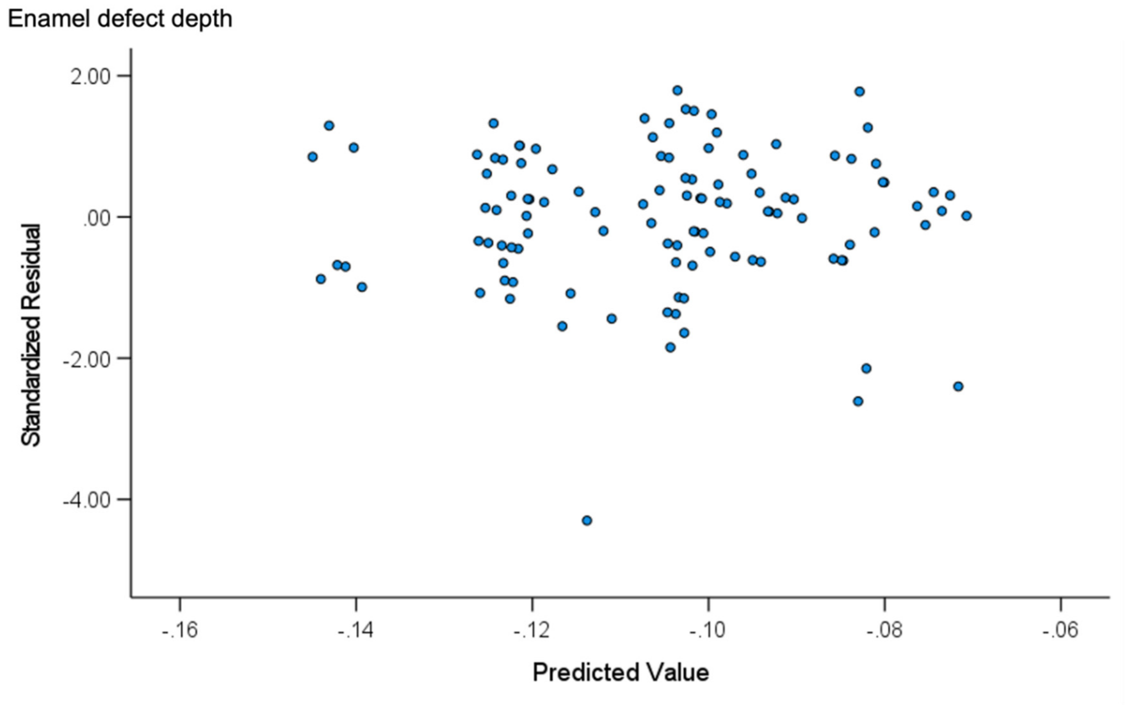
Enamel defect depth
2.00
.00
-2.00
-4.00
Standardized Residual
-.16
-.14
-.12
-.10
-.08
-.06
Predicted Value

(**c**)

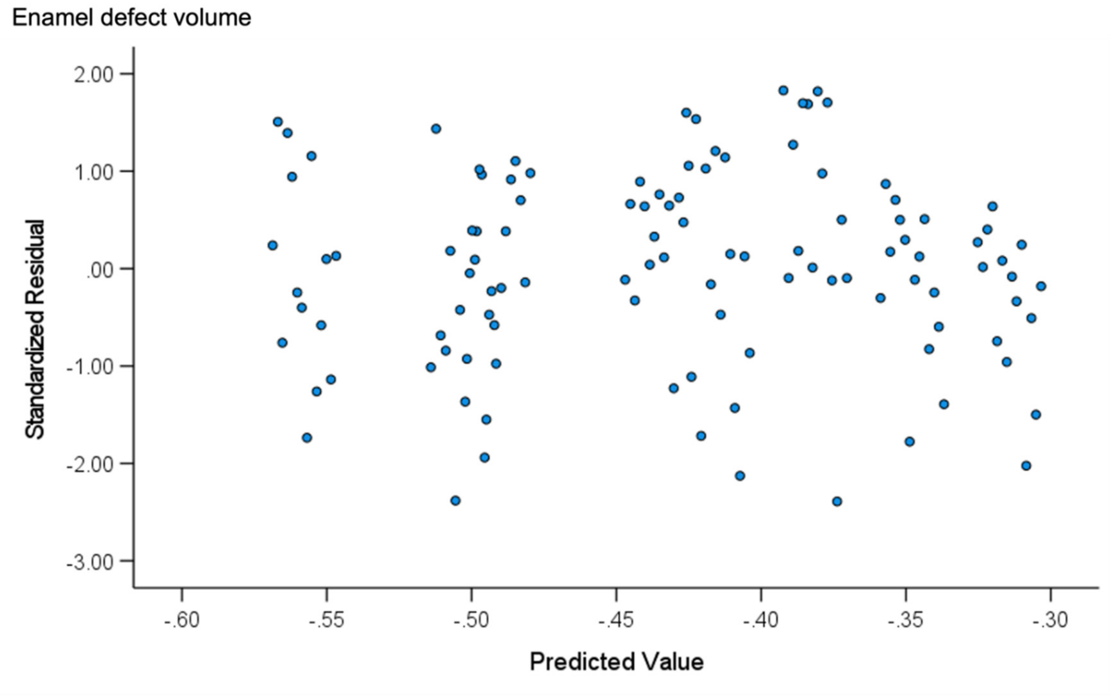
Enamel defect volume
2.00
1.00
.00
-1.00
-2.00
-3.00
Standardized Residual
-.60
-.55
-.50
-.45
-.40
-.35
-.30
Predicted Value

(d)

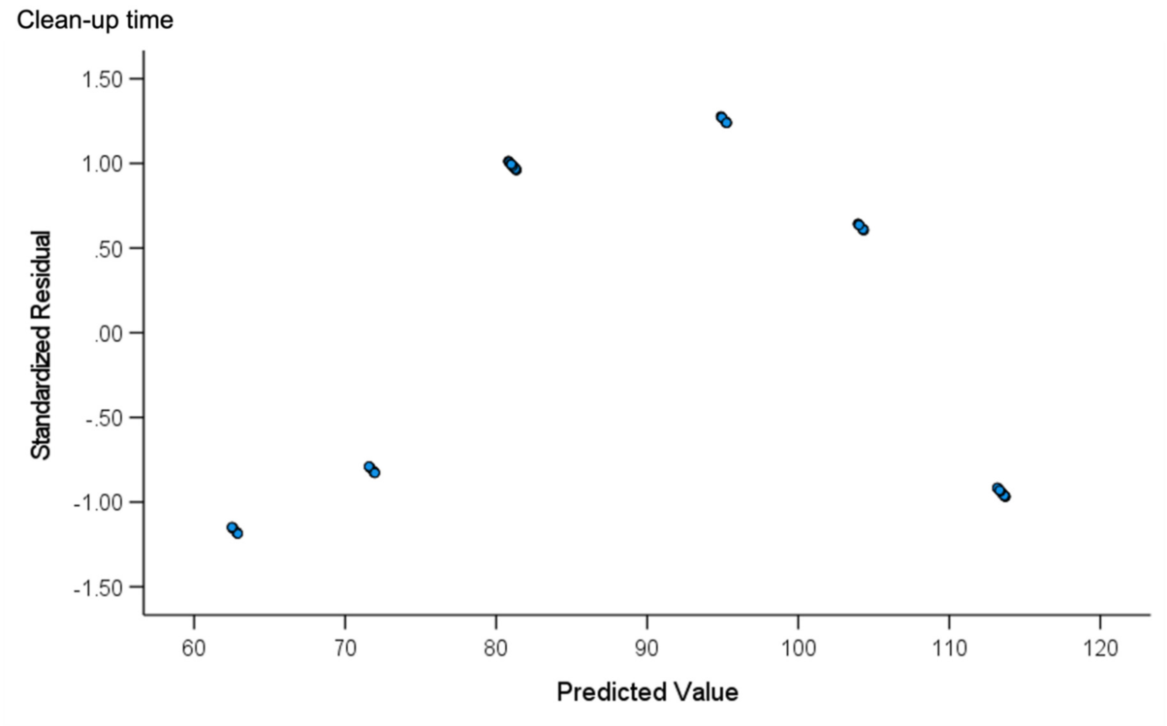
Clean-up time
1.50
1.00
.50
.00
-.50
-1.00
-1.50
Standardized Residual
60
70
80
90
100
110
120
Predicted Value

(e)
